# Supplementary material for: Analysis of the “naming game” with learning errors in communications
Source: Sci Rep. 2015 Jul 16;5:12191. doi: 10.1038/srep12191 (PMC4503979; doi:10.1038/srep12191)
Supplement: Supplementary Information [file srep12191-s1.doc]

Supplementary information for the manuscript

“Analysis of the “naming game” with learning errors in communications”

**Yang LOU** and **Guanrong CHEN***

Department of Electronic Engineering, City University of Hong Kong, Hong Kong SAR, China

*Corresponding author: eegchen@cityu.edu.hk

1. **On Small-world network with *K* = 40**

The networks of small-world *SW*/40/{0.1, 0.2, 0.3} are investigated in the following. The parameter settings are shown in Table S1, followed by the simulation results and statistical analysis.

1. Small-word network with number of neighborhoods *K* = 40 with different rewiring probabilities

| Notation | Network Type | Number of nodes | Average degree | Average path length | Average clustering coefficient |
| --- | --- | --- | --- | --- | --- |
| *SW*/40/0.1 | Small-world network with *K* = 40 and 𝑅𝑃 = 0.1 | 2,000 | 80.00 | 2.4499 | 0.5457 |
| *SW*/40/0.2 | Small-world network with *K* = 40 and 𝑅𝑃 = 0.2 | 2,000 | 80.00 | 2.2367 | 0.3894 |
| *SW*/40/0.3 | Small-world network with *K* = 40 and 𝑅𝑃 = 0.3 | 2,000 | 80.00 | 2.1291 | 0.2718 |

| 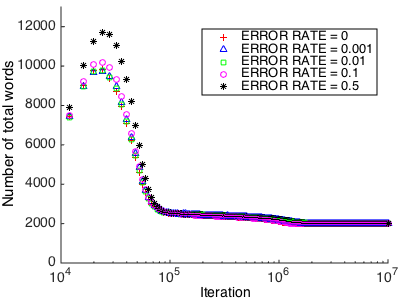 | 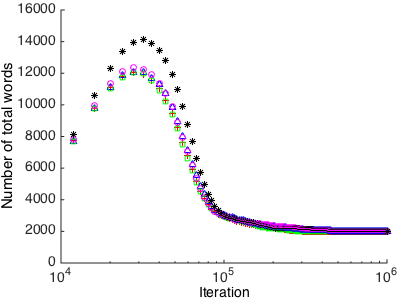 | 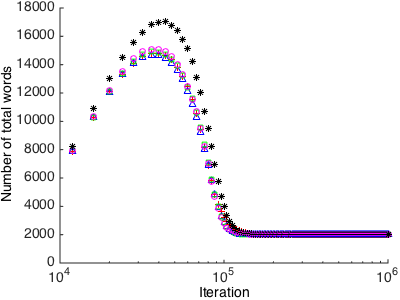 |
| --- | --- | --- |
| (a) *SW*/40/0.1 | (b) *SW*/40/0.2 | (c) *SW*/40/0.3 |

1. The convergence process in terms of the number of total words in small-world networks with the number of neighborhoods *K* = 40

| 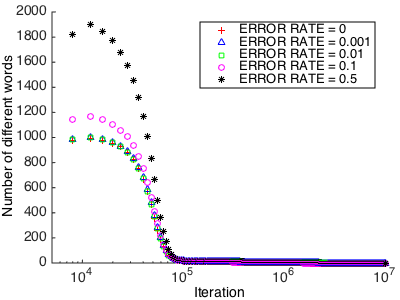 | 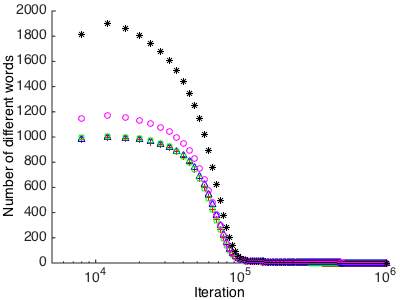 | 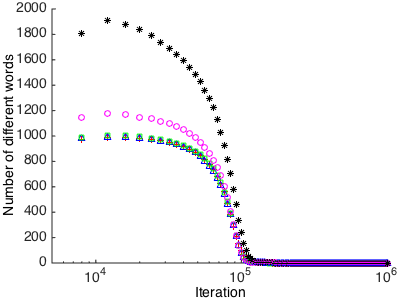 |
| --- | --- | --- |
| (a) *SW*/40/0.1 | (b) *SW*/40/0.2 | (c) *SW*/40/0.3 |

1. The convergence process in terms of the number of different words in small-world networks with the number of neighborhoods *K* = 40

| 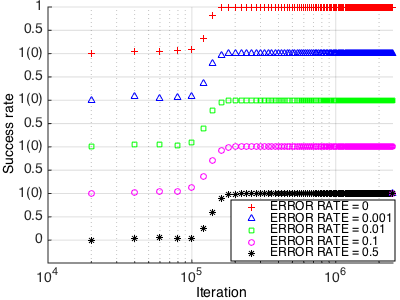 | 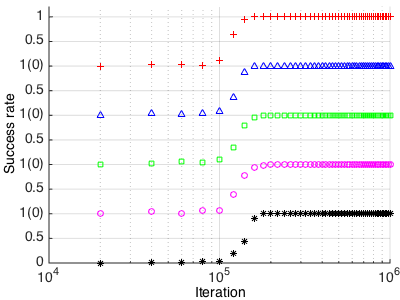 | 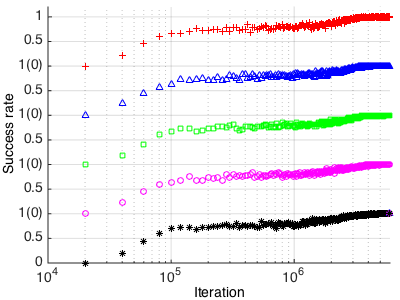 |
| --- | --- | --- |
| (a) *RG*/0.03 | (b) *RG*/0.1 | (c) *SW*/20/0.1 |
| 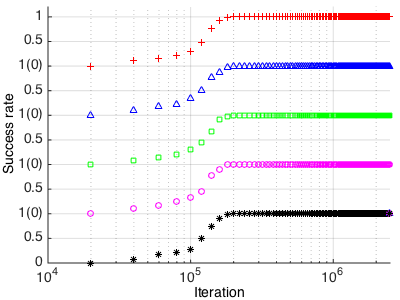 | 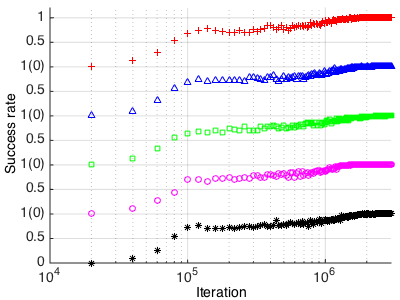 | 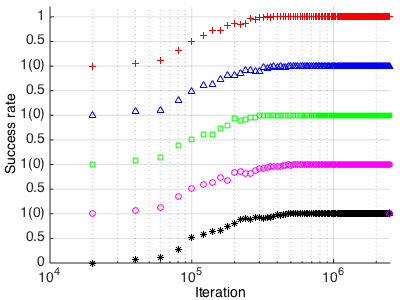 |
| (d) *SW*/20/0.3 | (e) *SW*/40/0.1 | (f) *SW*/40/0.2 |
| 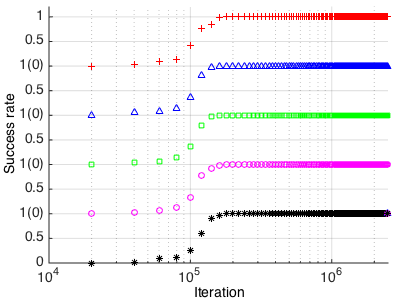 | 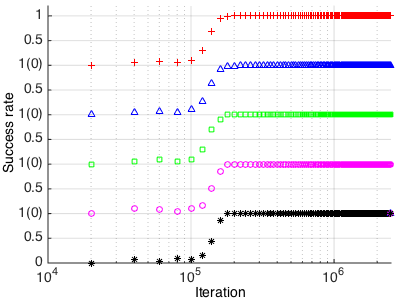 | 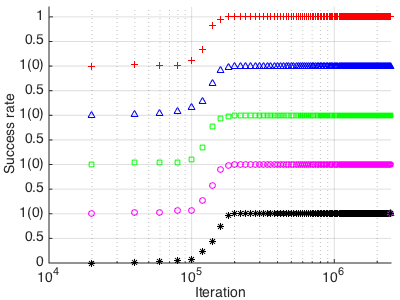 |
| (g) *SW*/40/0.3 | (h) *SF*/25 | (i) *SF*/75 |

1. Success rate curves including two random-graphs *RG*/0.03 and *RG*/0.1, five small-worlds *SW*/20/0.1, *SW*/20/0.3 and *SW*/40/{0.1, 0.2, 0.3}, and two scale-frees *SF*/25 and *SF*/75

| 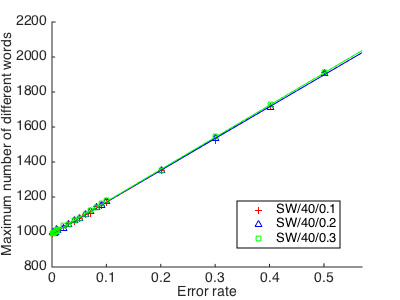 | 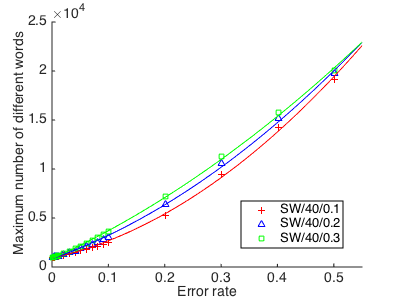 |
| --- | --- |
| (a) *SW*/40 | (b) *SW*/40 (without convergence) |

1. The relationship between the error rate and the maximum number of different words, in the topology of small-world with the number of neighborhoods *K* = 40, accompanied with (a) linear, and (b) quadratic fit curves for reference
2. The number of average convergence time, increment relationship between average convergence time with different values of the error rate (all the increments of convergence time are statistically insignificant in this table)

| Error  Rate  Network | 0 | 0.001 | 0.01 | 0.1 | 0.5 |
| --- | --- | --- | --- | --- | --- |
| *SW*/40/0.1 | 1.37E+06 | 1.30E+06 | 1.42E+06 | 1.47E+06 | 1.34E+06 |
| (Increment) | *NA* | -0.0522 | +0.0370 | +0.0709 | -0.0238 |
| *SW*/40/0.2 | 2.35E+05 | 2.53E+05 | 2.42E+05 | 2.29E+05 | 2.45E+05 |
| (Increment) | *NA* | +0.0783 | +0.0309 | -0.0249 | +0.0428 |
| *SW*/40/0.3 | 1.25E+05 | 1.22E+05 | 1.27E+05 | 1.19E+05 | 1.30E+05 |
| (Increment) | *NA* | -0.0243 | +0.0171 | -0.0499 | +0.0358 |


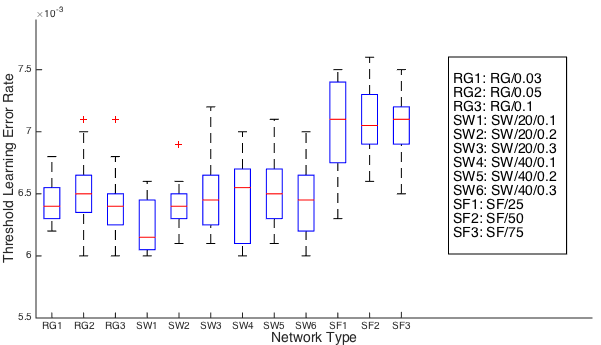


1. The box plot of learning error rate threshold in twelve types of networks with population size 2,000 in this study
2. **On population size**
3. Settings of networks with population sizes of 200 and 500

| Notation | Network type | Number of nodes | Average degree | Average path length | Average clustering coefficient |
| --- | --- | --- | --- | --- | --- |
| *RG*/0.05(0.2K) | Random-graph network with *P* = 0.1 | 200 | 19.91 | 2.0226 | 0.1001 |
| *RG*/0.05(0.5K) | Random-graph network with *P* = 0.1 | 500 | 24.96 | 2.2228 | 0.0500 |
| *SW*/20/0.2(0.2K) | Small-world network with *K* = 20 and 𝑅𝑃 = 0.2 | 200 | 40.00 | 1.8156 | 0.4334 |
| *SW*/20/0.2(0.5K) | Small-world network with *K* = 20 and 𝑅𝑃 = 0.2 | 500 | 40.00 | 2.1607 | 0.3966 |
| *SW*/40/0.2(0.2K) | Small-world network with *K* = 20 and 𝑅𝑃 = 0.2 | 200 | 80.00 | 1.5980 | 0.5162 |
| *SW*/40/0.2(0.5K) | Small-world network with *K* = 20 and 𝑅𝑃 = 0.2 | 500 | 80.00 | 1.8426 | 0.4240 |
| *SF*/50(0.2K) | Scale-free with 51 initial nodes and 50 new edges added at each step | 200 | 87.08 | 1.5633 | 0.5442 |
| *SF*/50(0.5K) | Scale-free with 51 initial nodes and 50 new edges added at each step | 500 | 94.80 | 1.8102 | 0.3085 |

In this section, we extent the proposed model naming game with learning errors in communications (NGLE) to different population size situations to check the scaling property of it. Four types of networks are studied here, including *RG*/0.05, *SW*/20/0.2, *SW*/40/0.2, and *SF*/50, the population size varies from 200, 500, 1,000, 2,000 to 3,000, where the population size 2,000 experimental results are presented in the manuscript, and the experimental results of population size 200 (denoted by (0.2K)), 500 (denoted by (0.5K)), 1,000 (denoted by (1K)), and 3,000 (denoted by (3K)) are presented as follows. The settings of networks with population sizes 200 and 500 are presented in Table S3, while the settings of the rest networks are shown in Table 1 of the manuscript and Table S1.

| 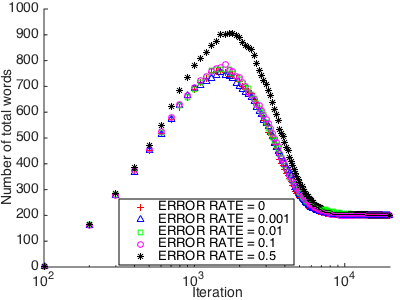 | 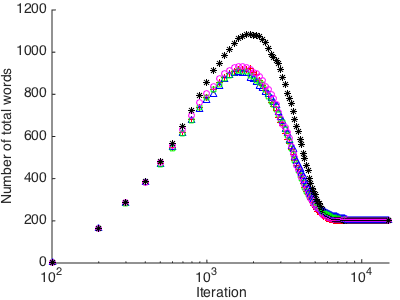 | 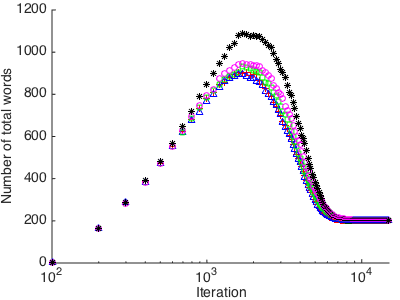 |
| --- | --- | --- |
| (a) *SW*/20/0.2(0.2K) | (b) *SW*/40/0.2(0.2K) | (c) *SF*/50(0.2K) |
| 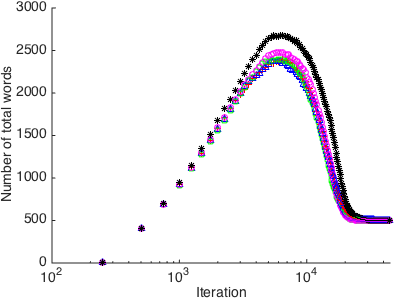 | 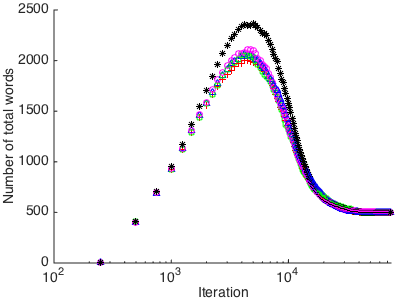 | 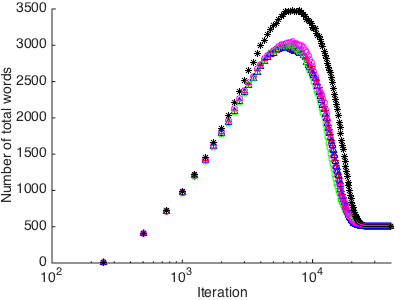 |
| (d) *RG*/0.05(0.5K) | (e) *SW*/20/0.2(0.5K) | (f) *SF*/50(0.5K) |
| 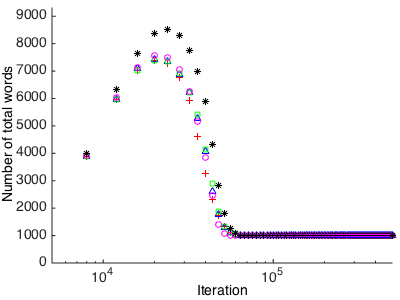 | 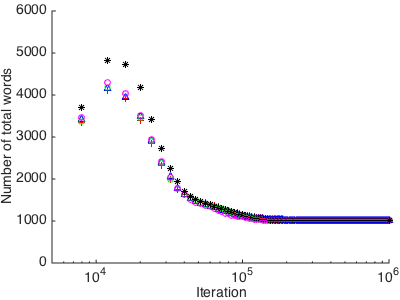 | 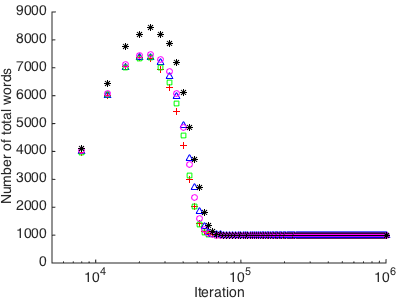 |
| (g) *RG*/0.05(1K) | (h) *SW*/20/0.2(1K) | (i) *SF*/50(1K) |
| 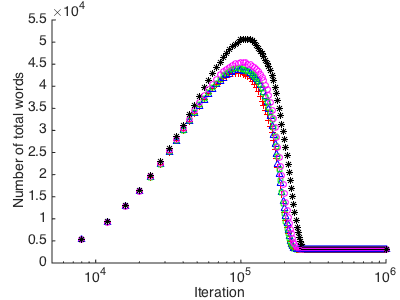 | 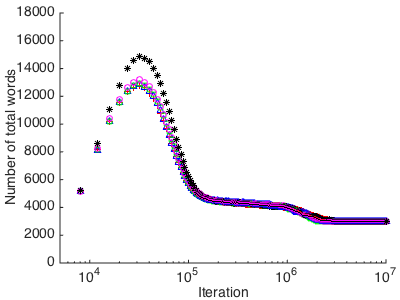 | 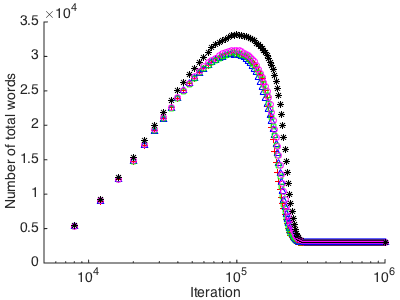 |
| (j) *RG*/0.05(3K) | (k) *SW*/20/0.2(3K) | (l) *SF*/50(3K) |

1. The convergence process in terms of the number of total words in a network with 200 nodes, 500 nodes, 1,000 nodes and 3,000 nodes, respectively

| 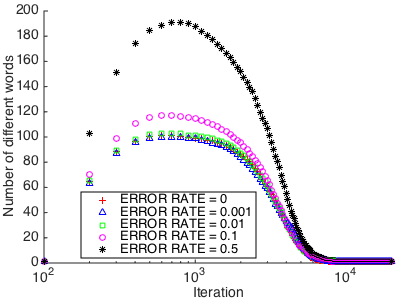 | 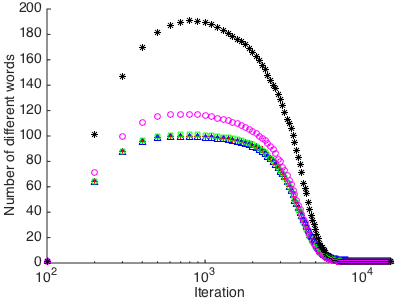 | 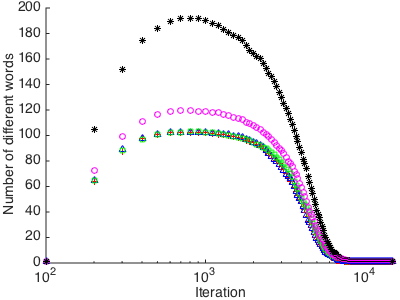 |
| --- | --- | --- |
| (a) *RG*/0.05 (0.2K) | (b) *SW*/20/0.2(0.2K) | (c) *SF*/50(0.2K) |
| 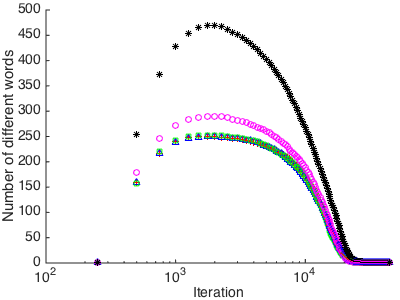 | 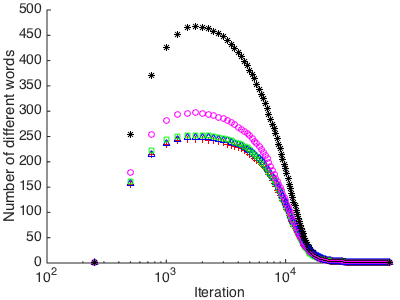 | 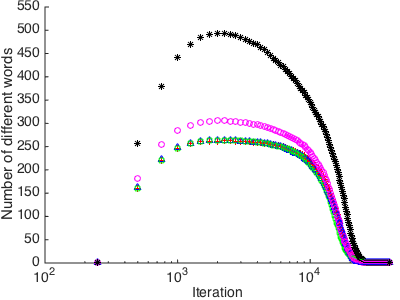 |
| (d) *RG*/0.05(0.5K) | (e) *SW*/20/0.2(0.5K) | (f) *SF*/50(0.5K) |
| 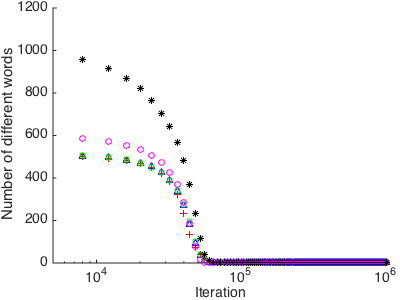 | 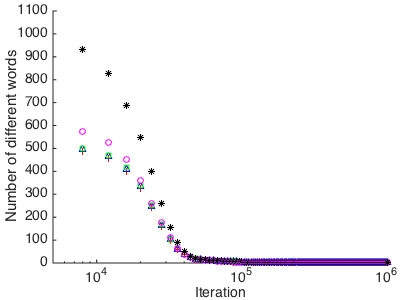 | 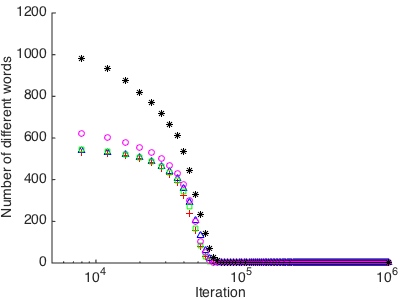 |
| (g) *RG*/0.05(1K) | (h) *SW*/20/0.2(1K) | (i) *SF*/50(1K) |
| 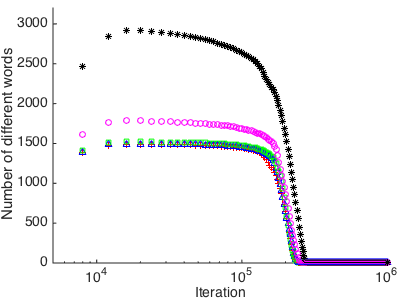 | 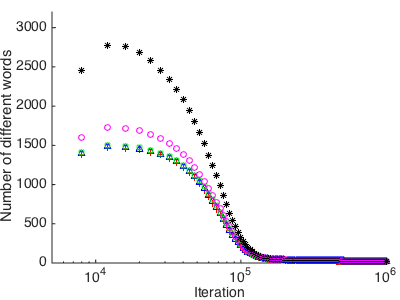 | 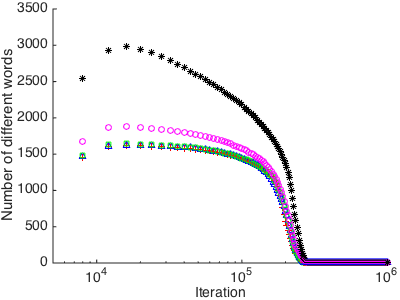 |
| (j) *RG*/0.05(3K) | (k) *SW*/20/0.2(3K) | (l) *SF*/50(3K) |

1. The convergence process in terms of the number of different words in a network with 200 nodes, 500 nodes, 1,000 nodes and 3,000 nodes, respectively

| 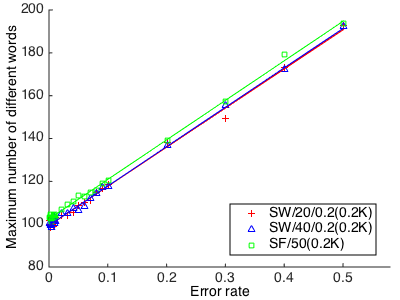 | 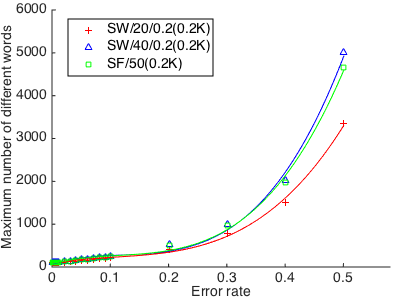 |
| --- | --- |
| (a) Three networks with 200 nodes | (b) Three networks with 200 nodes (without convergence) |
| 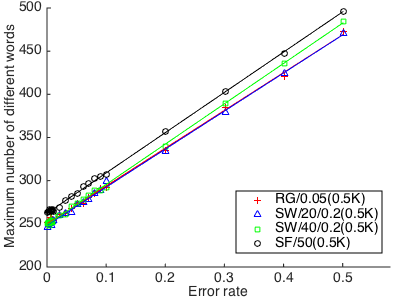 | 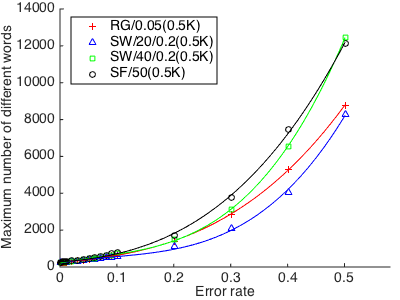 |
| (c) Three networks with 500 nodes | (d) Three networks with 200 nodes (without convergence) |

1. The relationship between the error rate and the maximum number of different words, on networks with population sizes of 200 and 500, respectively, with (a), (c) linear, and (b), (d) cubic fitted curves, used as reference
2. **On convergence time**

Tables S4 and S5 provides the accurate convergence time (based on the experimental results of population size 2,000), we set the convergence time for the case without learning errors as the benchmark, which is 1.0000 in the first row of the table, and in the following rows, the increment values of convergence time affected by different learning errors are shown. For example, +0.0116 (in row 2, column 1) means that, in the network with *RG*/0.03, when the error rate is 0.001, its convergence time increases by 0.0116 compared to that without learning errors, while with error rate 0.002, it requires only 0.9846 convergence time (−0.0154 in row 3, column 1) of that without learning errors.

In the cases of *random-graph* and *scale-free* networks, there exist some approximate thresholds, when error rates are greater than that the increments of convergence time become positive and relatively large. For the random-graph networks, the threshold of *RG*/0.03 is 0.4, for *RG*/0.05, it is 0.2, and for *RG*/0.1, it is 0.003. For the three scale-free networks simulated, they are 0.09, 0.09 and 0.06, respectively. In contrast, such threshold does not appear in *small-world* networks. There is not an observable relationship between error rates and increments of the convergence time. We denote the maximum positive/negative increments in bold for the results of *small-world* networks. The last row of Tables S4 and S5 summarizes the number of cases that increase and reduce the convergence time, respectively.

Table S6 gives the statistical numbers of different increment values, which reports that the most error-caused convergence delay are located within [0, 0.1), and the second is [−0.1, 0). Statistically, learning errors bring small increments to the convergence time.

1. The increment relationship between convergence time and different values of the error rate

| Net -works  Error  rate | *RG*/0.03 | *RG*/0.05 | *RG*/0.10 | *SF*/25 | *SF*/50 | *SF*/75 |
| --- | --- | --- | --- | --- | --- | --- |
| 0 | 1.0000 | 1.0000 | 1.0000 | 1.0000 | 1.0000 | 1.0000 |
| 0.001 | +0.0116 | +0.0490 | +0.0768 | +0.0232 | +0.0155 | +0.0431 |
| 0.002 | −0.0154 | +0.0770 | +0.0092 | +0.0412 | +0.0143 | +0.0098 |
| 0.003 | +0.0113 | +0.0455 | **+0.0828** | +0.0169 | −0.0428 | +0.0230 |
| 0.004 | −0.0121 | +0.0635 | +0.0751 | +0.0236 | −0.0121 | +0.0627 |
| 0.005 | −0.0213 | +0.0790 | +0.0653 | +0.0389 | +0.0802 | +0.0241 |
| 0.006 | −0.0175 | +0.0146 | +0.0898 | +0.0648 | +0.0062 | +0.0735 |
| 0.007 | −0.0502 | +0.0712 | +0.0788 | −0.0187 | +0.0486 | +0.0279 |
| 0.008 | −0.0609 | +0.0300 | +0.0579 | +0.0397 | +0.0165 | −0.0072 |
| 0.009 | −0.0166 | −0.0475 | +0.0962 | +0.0064 | −0.0079 | +0.0707 |
| 0.01 | +0.0193 | +0.0165 | +0.1122 | −0.0088 | −0.0228 | +0.0290 |
| 0.02 | −0.0301 | +0.0408 | +0.1176 | +0.0385 | −0.0129 | +0.0915 |
| 0.03 | +0.0255 | +0.0192 | +0.0863 | −0.0062 | +0.0114 | +0.0121 |
| 0.04 | +0.0152 | +0.0267 | +0.0979 | +0.0575 | +0.0560 | −0.0030 |
| 0.05 | −0.0356 | +0.0470 | +0.1305 | +0.0024 | −0.0019 | +0.0146 |
| 0.06 | +0.0425 | +0.0718 | +0.0917 | +0.0094 | +0.0266 | **+0.0557** |
| 0.07 | −0.0267 | +0.0320 | +0.0964 | −0.0051 | −0.0064 | +0.0638 |
| 0.08 | +0.0264 | +0.0249 | +0.1304 | −0.0050 | −0.0268 | +0.0500 |
| 0.09 | +0.0193 | −0.0007 | +0.0816 | **+0.0795** | **+0.0406** | +0.0271 |
| 0.1 | +0.0360 | +0.0205 | +0.1087 | +0.0556 | +0.0279 | +0.0689 |
| 0.2 | +0.0241 | **+0.0734** | +0.2003 | +0.0263 | +0.0260 | +0.0720 |
| 0.3 | +0.0383 | +0.1368 | +0.1524 | +0.0617 | +0.0887 | +0.0154 |
| 0.4 | **+0.0854** | +0.1609 | +0.1622 | +0.0332 | +0.0701 | +0.1406 |
| 0.5 | +0.0886 | +0.1719 | +0.2103 | +0.0473 | +0.0321 | +0.1265 |
| Summary | 13+/10− | 21+/2− | 23+/0− | 18+/5− | 15+/8− | 19+/2− |

1. The increment relationship between convergence time and different values of the error rate (continued)

| Net-works  Error Rate | *SW*/20/0.1 | *SW*/20/0.2 | *SW*/20/0.3 | *SW*/40/0.1 | *SW*/40/0.2 | *SW*/40/0.3 |
| --- | --- | --- | --- | --- | --- | --- |
| 0 | 1.0000 | 1.0000 | 1.0000 | 1.0000 | 1.0000 | 1.0000 |
| 0.001 | +0.0115 | +0.0935 | +0.0275 | +0.1785 | +0.1560 | −0.0376 |
| 0.002 | +0.0412 | −0.0392 | −0.0206 | +0.1827 | +0.0905 | +0.0004 |
| 0.003 | −0.0514 | −0.0955 | +0.0143 | +0.2084 | +0.1946 | −0.0433 |
| 0.004 | +0.0279 | −0.1055 | +0.0496 | +0.1518 | +0.1762 | −0.0077 |
| 0.005 | +0.0399 | **−0.1581** | +0.0144 | +0.1642 | +0.1546 | −0.0250 |
| 0.006 | +0.0001 | −0.0008 | +0.0062 | +0.1485 | +0.0026 | −0.0189 |
| 0.007 | **−0.0967** | −0.0935 | **+0.0666** | +0.1749 | +0.1534 | −0.0548 |
| 0.008 | +0.0242 | −0.0551 | +0.0377 | +0.0888 | +0.2110 | −0.0370 |
| 0.009 | −0.0297 | −0.0537 | +0.0017 | +0.1443 | **−0.0570** | −0.0636 |
| 0.01 | +0.0483 | −0.0040 | +0.0168 | +0.2334 | −0.0381 | −0.0516 |
| 0.02 | −0.0325 | −0.0668 | +0.0352 | +0.1067 | +0.0641 | −0.0073 |
| 0.03 | +0.0911 | −0.0595 | **−0.0513** | +0.0574 | +0.1501 | −0.0510 |
| 0.04 | +0.1052 | −0.1037 | +0.0385 | +0.0925 | **+0.2737** | **+0.0284** |
| 0.05 | **+0.1472** | +0.0461 | +0.0419 | +0.1268 | +0.1548 | −0.0384 |
| 0.06 | −0.0162 | +0.0484 | +0.0297 | +0.1356 | +0.0809 | −**0.0868** |
| 0.07 | −0.0155 | −0.1183 | +0.0592 | +0.1527 | +0.2576 | −0.0611 |
| 0.08 | −0.0280 | −0.0843 | +0.0090 | **+0.2858** | +0.1552 | 0.0145 |
| 0.09 | −0.0799 | **+0.1211** | +0.0359 | +0.1217 | +0.0554 | −0.0054 |
| 0.1 | +0.0260 | +0.0262 | −0.0078 | **−0.0002** | +0.2330 | −0.0312 |
| 0.2 | −0.0034 | +0.0769 | +0.0383 | +0.1560 | +0.1912 | −0.0125 |
| 0.3 | +0.1204 | +0.0074 | −0.0193 | +0.1110 | +0.2616 | −0.0060 |
| 0.4 | +0.0430 | −0.0764 | +0.0449 | +0.0558 | −0.0328 | −0.0194 |
| 0.5 | −0.0334 | −0.0604 | +0.0009 | +0.2607 | +0.1551 | +0.0209 |
| Summary | 13+/10− | 7+/16− | 19+/4− | 22+/1− | 20+/3− | 4+/19− |

1. Statistical division of the numbers of different incremental values in Tables S4 and S5 into different intervals

| Increment value intervals | (−∞, −0.2) | [−0.2, −0.1) | [−0.1, 0) | [0, 0.1) | [0.1, 0.2) | [0.2, +∞) |
| --- | --- | --- | --- | --- | --- | --- |
| Number of data | 0 | 4 | **76** | **145** | 40 | 11 |
